# A Novel Information-Theoretic Approach for Variable Clustering and Predictive Modeling Using Dirichlet Process Mixtures

**DOI:** 10.1038/srep38913

**Published:** 2016-12-14

**Authors:** Yun Chen, Hui Yang

**Affiliations:** 1 School of Mechanical Engineering, Jiangsu University of Science and Technology, Zhenjiang, China; 2 Complex Systems Monitoring, Modeling and Control Laboratory, The Pennsylvania State University, University Park, PA, USA

## Abstract

In the era of big data, there are increasing interests on clustering variables for the minimization of data redundancy and the maximization of variable relevancy. Existing clustering methods, however, depend on nontrivial assumptions about the data structure. Note that nonlinear interdependence among variables poses significant challenges on the traditional framework of predictive modeling. In the present work, we reformulate the problem of variable clustering from an information theoretic perspective that does not require the assumption of data structure for the identification of nonlinear interdependence among variables. Specifically, we propose the use of mutual information to characterize and measure nonlinear correlation structures among variables. Further, we develop Dirichlet process (DP) models to cluster variables based on the mutual-information measures among variables. Finally, orthonormalized variables in each cluster are integrated with group elastic-net model to improve the performance of predictive modeling. Both simulation and real-world case studies showed that the proposed methodology not only effectively reveals the nonlinear interdependence structures among variables but also outperforms traditional variable clustering algorithms such as hierarchical clustering.

Predictive modeling extracts useful information and patterns from the data to drive decisions or actions. For example, insurance companies have gathered a vast amount of data in their data warehouses^1^. The objective of the predictive model is not only to improve the pricing or marketing process, but also to analyze profitability, fraud, catastrophe, and other insurance operations. In the 21st century, wireless sensing, electronic health records, and health Internet of Things are increasingly adopted to assist in the process of clinical decision making^2^,^3^,^4^. This amount of information from multiple sources provides numerous variables for the contemplated predictive model.

When a predictive model involves large amounts of variables (i.e., explanatory or response variables), researchers are confronted with the need to reduce the number of variables in order to build the compact model. To some extent, the variables are unknown to be redundant or relevant to the objective of predictive models but rather need to be tested with real-world data. In addition, when there is an enormous amount of variables, it becomes difficult to find out the relationship between variables. If the model building involves too many variables, it will impact the model compactness and efficiency. There is also a possibility to increase the model sensitivity to noises and overfit the data with many variables. The model parameters are not stable when variables are highly correlated. It is even more difficult to explain the physical meanings of the predictive model when there are many variables. Finally, model building with a large amount of variables is computationally expensive and could take indefinite time for the exhaustive search. An intermediate approach to the exhaustive search may also be time-consuming and some combinations of variables could be overseen.

Data clustering is an unsupervised method to group data samples into homogeneous clusters, while variable clustering is to detect subsets of homogeneous variables and then cluster them into the same group, in which variables have stronger interrelations to each other than to those in other groups. As shown in Fig. 1a, data clustering groups data samples into clusters and each sample has two values, e.g., (0.26, −0.09) in the 2-dimensional space, where X-axis is the value of variable 1, and Y-axis is the value of variable 2. Data samples are clustered based on the similarity measure, e.g., Euclidean distance. However, variable clustering is different from data clustering. Figure 1b illustrates the clustering results of 15 variables, each of which has 1000 data samples. For example, the variable 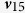 represents a series of 1000 samples. Notably, each point in Fig. 1b is a variable instead of a data sample.

Variable clustering uncovers natural groups of objects (variables, features, or factors) in a multivariate dataset. The hierarchical clustering (HC)^5^, a generic clustering procedure, sequentially merges pairs of clusters that share common characteristics based on similarity measures. HC procedures generate a nested set of partitions, also called hierarchy. The choice of the similarity measure plays an important role in the clustering process because it indirectly defines the structure of the clusters. This choice is not only guided by problems to solve, but also restricted to commonly used measures, such as the Euclidean distance or Pearson’s correlation coefficient. However, nonlinear interdependence among variables cannot be adequately captured by linear correlation. Further, we cannot relocate the variables once the merge is done for two closest clusters, because HC is not a dynamic approach. There is no adaptive step for two variables to make modifications in the later stage if they are ‘incorrectly’ clustered at the early stage.

In this paper, we develop a new methodology of information theoretic approach for variable clustering and predictive modeling. The proposed approach investigates both redundancy and relevancy among variables. Specifically, nonlinear interdependence structures are measured among variables. Further, we introduced nonparametric Dirichlet process to cluster embedded variables with their probability distributions. Finally, orthonormalized variables were integrated with group elastic net models to improve the performances of predictive models. Both simulation and real-world case studies demonstrate that the proposed methodology not only outperforms traditional variable clustering algorithms such as hierarchical clustering, but also effectively identifies nonlinear interdependence structures among variables and further improves the performance of predictive modeling.

## Research Background

### Clustering Analysis

**Figure 2:**
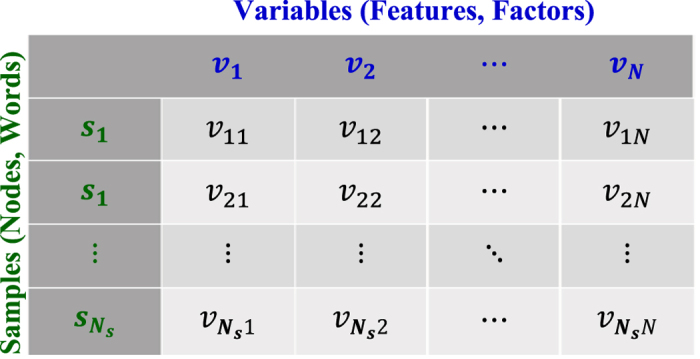
Data in the table form, where variables are in columns and samples are in rows.

When “clustering” is used in the literature, it is referred to be “data clustering” for most of the time. The approach of data clustering groups data samples into homogeneous subsets, in which data samples are closer to each other in the same cluster than to other clusters. As shown in Fig. 2, data clustering is more concerned about the samples that are rows (i.e., 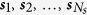) in the table format of a dataset but variable clustering focus on the variables in the columns (i.e., ***v***_1_, ***v***_2_, …, ***v***_*N*_). The variables, ***v***_1_, ***v***_2_, …, ***v***_*N*_, are also known as features or factors, where 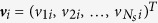, 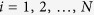, *N* is the number of variables and *N*_*s*_ is the number of samples respectively. The samples, 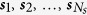, are also called nodes in the network or words in the text, where ***s***_*j*_ = (*v*_*j*1_, *v*_*j*2_, …, *v*_*jN*_)^*T*^, *j* = 1, 2, …, *N*_*s*_. It may be noted that big data often brings a large number of variables that can be bigger than the number of samples, i.e., *N > N*_*s*_. Complex interdependence structures among variables significantly challenge the traditional framework of predictive modeling. As such, variable clustering to delineate homogeneous groups of variables is urgently needed.

In recent years, community detection in network analysis receives increasing interests in data clustering. Network-based methods cluster nodes with strong connections into a community. For example, mixed membership stochastic blockmodels (MMSB)^6^ were proposed to discover complex network structure in a variety of applications, e.g., large-scale protein interaction network and social network. The MMSB develops a novel class of latent variable models for relational data, and assumes each variable belongs to multiple communities/clusters rather than a single community/cluster. Joint Gamma process Poisson factorization (J-GPPF)^7^ was developed to jointly model sparse networks with large size and side information. Infinite edge partition model^8^ was introduced to not only study overlapping communities and inter-community interactions but also predict missing edges. However, community detection groups nodes that represent data samples (e.g., proteins), rather than variables, into communities by considering the unweighted or weighted edges between nodes.

In addition, topic models are widely used for data clustering in the field of text mining. Topic models are statistical models for discovering topics that occur in a collection of documents with a large number of words (i.e., data samples in rows of table-form data in Fig. 2). Latent Dirichlet allocation (LDA)^9^ was first introduced as an unsupervised model to cluster documents in the topic space. LDA assumes the topic distribution to have a Dirichlet prior and maximizes the likelihood (or posterior probability) of the document collection. It may also be noted that supervised topic models with side information (e.g., document categories or review rating scores) were proposed to find latent topics and provide more predictive power than regression on unsupervised LDA features. For example, supervised latent Dirichlet allocation (sLDA)^10^ introduced the real-valued document rating as regression response and jointly modeled the documents and response by maximizing the joint likelihood. Maximum entropy discrimination LDA (MedLDA)^11^,^12^ proposed a unified constrained optimization framework that solves problems of dimensionality reduction and max-margin classification using features in the reduced-dimension space. Topic models formulate statistical models based on the intuition that specific words would appear more or less frequently in the document for a given topic. However, variable clustering does not hold this intuition. As such, topic models address special clustering problems in text mining that are different from other general data clustering or variable clustering problems.

Moreover, many previous approaches group a dataset into co-clusters (or biclusters), which are subsets of data samples exhibit similar behaviors across a subset of variables, or vice versa. Co-clustering approaches have been widely used in a variety of applications such as biological gene expression data^13^ and text mining^14^,^15^. Notably, a simultaneous co-clustering and learning (SCOAL)^16^ framework was proposed to generalize co-clustering and construct predictive models simultaneously. The SCOAL co-cluster the entire dataset into subsets of samples and variables such that each subset can be well characterized by a predictive model. However, the whole data set is divided into multiple subsets that capture incomplete data information. These subsets are then used to construct multiple predictive models rather than one model. In addition, nonlinear correlations among variables were not fully utilized in traditional co-clustering approaches. Instead, nonlinear predictive models were usually introduced to account for data nonlinearity, which also brings a large number of parameters.

### Hierarchical Clustering

Variable clustering is the task to group homogeneous variables into the same category, in which variables have stronger interrelations than to those in other groups. Variable clustering considers the interdependence structure among variables, e.g., correlation. The Pearson’s correlation^17^ between variables ***v***_1_ and 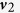 is


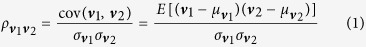


where cov(***v***_1_, ***v***_2_) is the covariance between ***v***_1_ and ***v***_2_, 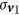 and 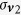 are variances of ***v***_1_ and ***v***_2_, 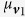 and 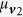 are means of ***v***_1_ and 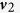, *E* is the expectation. However, the Pearson’s correlation only measures the linear relationship between variables ***v***_1_ and ***v***_2_.

In the literature, Pearson’s correlation was integrated with hierarchical clustering (HC) for variable clustering^5^. There are two ways to perform HC procedures - the agglomerative way and the divisive way. For example, agglomerative HC defines each variable as a singleton cluster in the first step. Then, two closest clusters with smallest dissimilarity measure are merged into one cluster. The merging process recursively moves up along the hierarchy until the stopping criterion is satisfied, e.g., the maximum number of clusters or the maximum group-average (GA) dissimilarity. The criterion of group average measures the average intergroup dissimilarity between two clusters, i.e.,


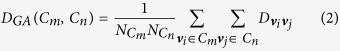


where 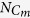 and 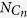 are the sizes of cluster *C*_*m*_ and *C*_*n*_, 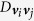 is the dissimilarity between variables ***v***_*i*_ and ***v***_*j*_, which is usually calculated as 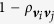.

**Figure 3:**
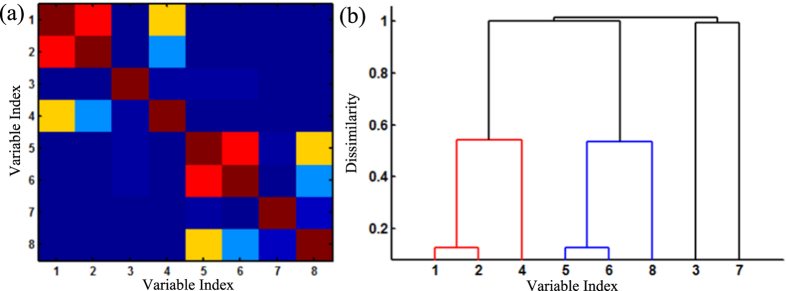
Illustration of Pearson’s correlation (**a**) and hierarchical clustering (**b**).

Here, a motivating example is shown to evaluate the performance of HC with Pearson’s correlation for variable clustering. Two clusters of variables are generated as follows:


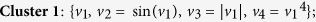



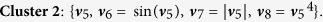


where ***v***_1_ and ***v***_5_ are independent standard normal variables. In the cluster 1, variable ***v***_1_ has linear correlation with variable ***v***_2_ and nonlinear correlation with variables ***v***_3_ and ***v***_4_. The cluster 2 has the similar situation. Figure 3a shows the correlation matrix of these eight variables. The red color represents a high correlation, while the blue color indicates no interrelationships. It may be noted that the correlation matrix effectively detects the linear correlation between variables ***v***_1_ and ***v***_2_, ***v***_5_ and ***v***_6_. However, nonlinear correlations are not well captured. Figure 3b shows the hierarchical clustering results based on the Pearson’s correlation. Variables ***v***_1_, ***v***_2_ and ***v***_4_ are clustered in the same cluster, and variables ***v***_5_, ***v***_6_ and ***v***_8_ are clustered in another cluster. However, hierarchical clustering failed to cluster variable ***v***_3_ into Cluster 1, and variable ***v***_7_ into Cluster 2. This is mainly due to the fact that nonlinear correlations among variables are not fully considered. Very little work has been done to cluster a large number of variables with complex structures of nonlinear interdependences. Thus, we propose a new methodology that integrates information theoretic approach with Dirichlet process mixtures for variable clustering and predictive modeling.

### Research Methodology

In this section, we will first characterize nonlinear correlation (i.e., mutual information) among variables and then embed variables in the lower-dimensional space. Second, we introduce the nonparametric Dirichlet process (DP) to derive self-organizing clusters of homogeneous variables with specific consideration of nonlinear interdependence. Finally, we orthonormalize variables in each cluster and then integrate them with group elastic-net model to improve the performance of predictive modeling.

### Mutual Information based Embedding of Variables

First, mutual information is characterized and quantified among variables. Traditionally, such interrelationships are estimated with linear methods such as Pearson’s correlation. As aforementioned, Pearson’s correlation, a second-order quantity, evaluates merely linear dependency among data and is limited in the ability to represent the variable-to-variable dissimilarities. Therefore, we propose to characterize the variable-to-variable dissimilarity matrix using mutual information and further embed variables into low-dimensional feature vectors that preserve the dissimilarity distances among variables.

Mutual information^18^ quantifies both linear and nonlinear interdependence between two variables ***v***_*i*_ and ***v***_*j*_, i.e., *i*th and *j*th columns in Fig. 2. Although there are various measures that capture nonlinear correlations among variables, mutual information has the advantage to equitably quantify statistical associations between two variables that is insensitive to the form of the underlying function^19^, where equitability means that the statistic gives similar scores to equally noisy relationships of different types^20^. In other words, mutual information has an attractive feature to provide an equitable measure of association between two variables that is insensitive to the form of the underlying function^19^. It may be noted that mutual information was introduced to cluster nonlinear structures among data samples (e.g., feature vectors of a gene, a company and a movie) by formulating a tradeoff function among average similarity and information carried by the cluster identities^21^. However, this information-theoretic approach considers nonlinear correlation structures among data samples, rather than variables, by introducing mutual information as a similarity measure. Moreover, the number of clusters was pre-defined in order to solve the tradeoff function.

**Figure 4:**
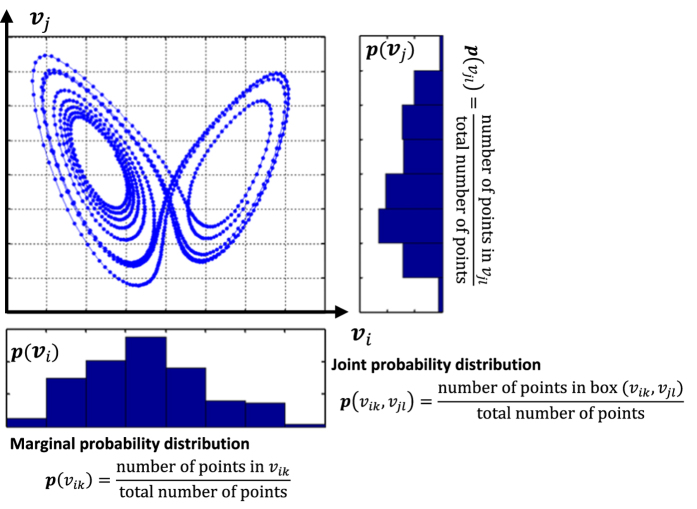
An illustration for the computation of mutual information.

The mutual information is defined as:


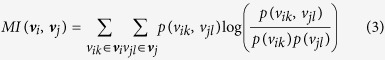


where *p*(*v*_*ik*_, *v*_*jl*_) is the joint probability distribution, *p*(*v*_*ik*_) and *p*(*v*_*jl*_) are marginal probabilities. Figure 4 shows the practical implementation to compute the mutual information with the scatter plot of two variables ***v***_*i*_ and ***v***_*j*_, and the marginal histogram for each variable. Marginal probabilities *p*(*v*_*ik*_) and *p*(*v*_*jl*_) are computed as the number of points in *v*_*ik*_ and *v*_*jl*_ divided by the total number of points in the 2-dimensional space. While the joint probability *p*(*v*_*ik*_, *v*_*jl*_) is computed as the number of points in box (*v*_*ik*_, *v*_*jl*_) divided by the total number of points in the space. In practice, large box size will lead to an accurate estimation of average probability, but a flat estimation of joint probability *p*(*v*_*ik*_, *v*_*jl*_). As such, this will underestimate the mutual information *MI*(***v***_*i*_, ***v***_*j*_). In contrast, small box size estimates the joint probability *p*(*v*_*ik*_, *v*_*jl*_) in small scales but brings significant variations, which overestimate the mutual information *MI*(***v***_*i*_, ***v***_*j*_). In the present investigation, we choose the number of bins as 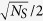
21, where *N*_*S*_ is the sample size.

Once the mutual information is computed for each pair of variables, the dissimilarity matrix among variables will be generated. It may be noted that the mutual information is inversely proportional to the dissimilarity. Therefore, we define *δ*_*ij*_ = 1/*MI*(***v***_*i*_, ***v***_*j*_) as the dissimilarity measure between *i*th and *j*th variables in *N* × *N* dissimilarity matrix Δ. Further, an embedding algorithm is developed to transform the dissimilarity matrix into low-dimensional feature vectors that preserve the variable-to-variable dissimilarity matrix. Let ***y***_*i*_ and ***y***_*j*_ denote the *i*th and *j*th feature vectors. The objective function is formulated as:


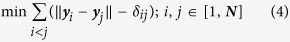


where ||·|| is the Euclidean norm. The Gram matrix *B* is firstly reconstructed from the dissimilarity matrix Δ in order to solve this optimization problem:


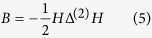


where *H* = *I* − *N*^−1^11^*T*^ is the centering matrix, *I* is the identity matrix with size *N* and 1 is a column vector with *N* ones. The Δ^(2)^ is a squared matrix and each element in Δ^(2)^ is 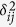. Then the element *b*_*ij*_ in matrix B is:


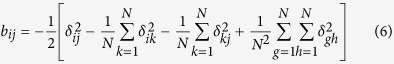


Due to the property of Gram matrix, it is defined as the scalar product *B* = *YY*^*T*^, where the matrix *Y* minimizes the aforementioned objective function. It is known that Gram matrix *B* is decomposed as:


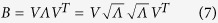


where *V* is a matrix of eigenvectors and Λ is a diagonal matrix of eigenvalues. Then, the matrix of feature vectors is obtained as: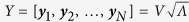. As such, each variable is embedded as a feature vector in the low-dimensional network that preserves the dissimilarity matrix.

### Dirichlet Process for Variable Clustering

Furthermore, we propose to cluster low-dimensional feature vectors that are embedded from variables. Although K-Means clustering is the most popular algorithm for data clustering^22^, it has several drawbacks. First, it is a parametric model and the number of clusters needs to be predefined. For clusters that are not well separated, this may not be straightforward. Second, K-Means algorithm needs to recalculate the objective function for assigning a cluster label to a new variable. Third, the results of K-Means clustering are not unique due to the recalculation of objective function. Therefore, we introduced the nonparametric Dirichlet process (DP) models to cluster variables^23^,^24^. DP models partition the vector space into local clusters, and assign cluster labels for new observations according to the assignment probability derived from the mean and covariance of each cluster, with each one following a multivariate Gaussian distribution.

**Figure 5:**
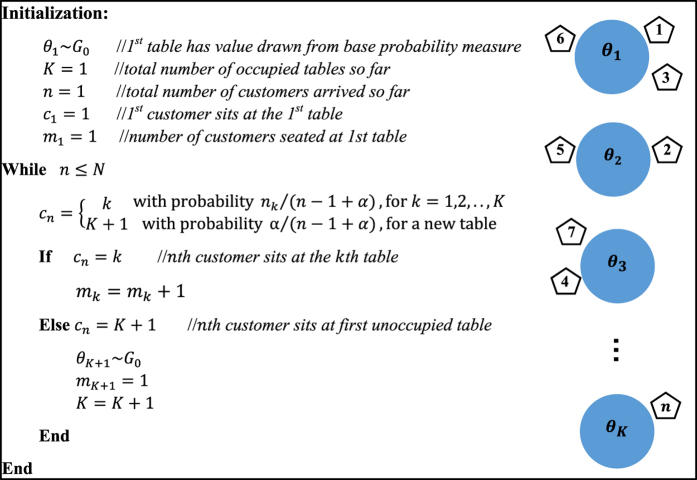
Algorithm and illustration of Chinese Restaurant Process.

The Chinese Restaurant Process (CRP) is an effective representation of DP, which visualizes the clustering effects more explicitly. Figure 5 shows the algorithm and illustration of CRP. Suppose a restaurant has potentially infinite many tables *k* = 1, 2, …, and each table has value *θ*_*k*_ drawn from base probability measure *G*_0_. Customers are indexed by *n* = 1, 2, …, *N* as they arrive, while indicator variables *c*_*n*_ = *k* denotes that the *n*th customer choose to sit at the *k*th table. The tables are chosen according to the following random process:

1. The first customer always chooses the first table.
2. The *n*th customer chooses an existing *k*th table with probability *m*_*k*_/(*n* − 1 + *α*), and a new table with probability α/(*n* − 1 + *α*).

where *α* > 0 is a concentration parameter, and *m*_*k*_ denotes the number of customers seated at the *k*th table. From the conditional probability distribution above, we can see that a customer is more likely to sit at a table if there are already many people sitting there. However, a customer will sit at a new table with the probability proportional to *α*.

This CRP provides an effective representation for the inference in Dirichlet process mixture models (DPMM). In DPMM, the distribution of indicator variables *c*_1_, *c*_2_, …, *c*_*N*_ given mixing proportions ***π*** = (*π*_1_, *π*_2_, …, *π*_*K*_) is multinomial


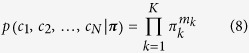


where 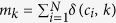 is the number of data points in *k*th cluster and 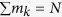. Since the Dirichlet distribution is conjugate to the multinomial, we can assume mixing proportions ***π*** for *K* clusters have a Dirichlet prior


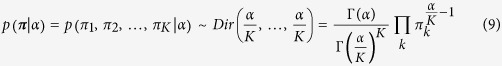


Then, integrating out the mixing proportions gives:


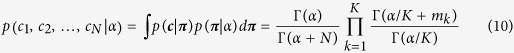


If the total number of clusters *K* is finite, then the probability of *n*th data point belongs to *k*th cluster given all other data points and concentration parameter *α* is


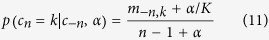


where *c*_−*n*_ denotes all indices except *n*, and *m*_−*n*,*k*_ = ∑_*i* ≠ *n*_*δ*(*c*_*i*_, *k*) is the number of data points in the *k*th cluster for assigning the first (*n* − 1) data points. If *K* is infinite as *K* → ∞, we can update the posterior indicator distribution using Gibbs sampling as:


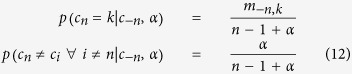


The distribution for a new variable y_*_ within a mixture cluster follows normal distribution


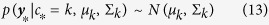


where the parameters *μ*_*k*_ and Σ_*k*_ are the mean and the covariance for cluster *k*. As a result, the weight for each cluster is obtained as


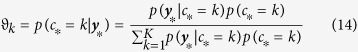


Due to the nonparametric nature of DP, the shape as well as the number of clusters need not be known a priori. Therefore, DP clusters are derived from characteristics inherent to data.

### Predictive Modeling with Clustered Variables

Although the Dirichlet process clusters variables into different groups, the variables in each group are similar to each other and thus bring the redundant information. It is necessary to delineate the structure of latent variables hidden in each cluster. As such, homogeneous variables in each cluster are orthonormalized before predictive modeling. Assume we have *K* clusters and there are 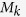 variables, i.e., 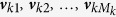, in the *k*-th cluster. Then, the redundant information within original variables 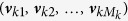 is minimized by transforming them into the orthonormal set of new variables 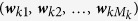 in each cluster using the Gram-Schmidt orthonormalization (GSO). The procedure begins by normalizing 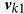,


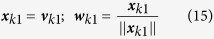


where ***w***_*k*1_ is the normalized variable of ***v***_*k*1_. Then, we orthogonalize and normalize the second vector ***v***_*k*2_ as,


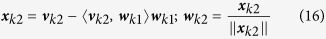


where ***w***_*k*2_ is the second orthonormalized vector. The process is recursively updated to get the *m*-th orthogonal vector ***x***_*km*_


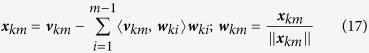


where ***w***_*km*_ is the *m*-th orthonormalized vector. Further, we leverage orthonormalized variables in each cluster to develop a group elastic-net model^25^, which achieves the model sparsity by the group-level and individual-level selection of features. The elastic net criterion is defined as:


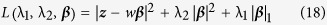


where λ_1_ and λ_2_ are non-negative real values. The elastic net estimator 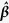 is to minimize the equation


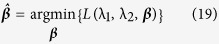


Solving 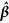 is equivalent to the optimization problem


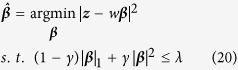


where 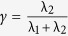 and *λ* is the tuning parameter.

To develop the group elastic-net model for logistic regression, we define *h*_***β***_(***w***, *i*) as the probability for z_*i*_ being a success (i.e., *z*_*i*_ = 1) and thus 1 − *h*_***β***_(***w***, *i*) is the probability for *z*_*i*_ being a failure (i.e., *z*_*i*_ = 0), where 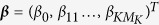 is the coefficient vector. Then we have


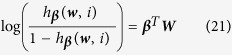


and


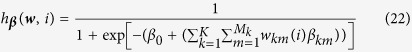


The likelihood function given observations (***w***(*i*), *z*_*i*_) is


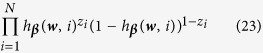


Taking the logarithm for equation, we have


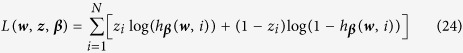


Therefore, we derive the group elastic-net model as:


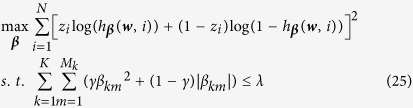


where *γ* and *λ* are penalization parameters, the logistic function *h*_***β***_(***w***, *i*) is used in the likelihood function because of the binary responses. The proposed approach will be evaluated and validated using experimental studies. The details are shown in the next section.

## Experimental Materials and Results

In this section, we evaluate and validate the proposed methodology using both simulation and real-world case studies.

### Simulation Study

First, a simulation study is shown to evaluate the performance of the proposed methodology for variable clustering. We simulate four clusters of variables in Table 1 as follows.

**Figure 6:**
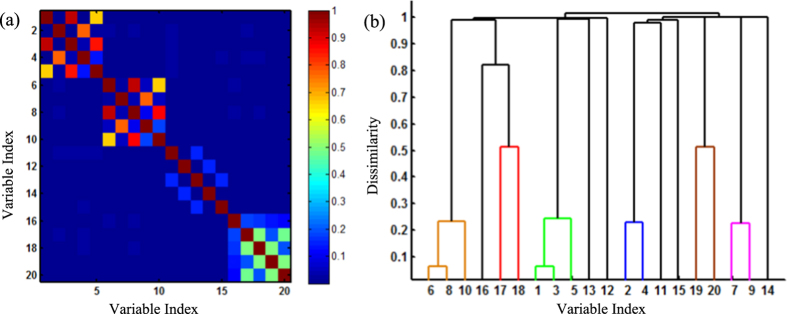
(**a**) Matrix of Pearson’s correlation; (**b**) Hierarchical clustering of simulated variables.

Figure 6a shows the matrices of Pearson’s correlations among variables that are computed from the simulation data set. Notably, the linear correlation in Fig. 6a cannot fully identify the nonlinear interdependence among simulated variables. Figure 6b shows that the HC cannot delineate the cluster structure of variables. This is mainly due to the fact that Pearson’s correlation is limited in the ability to detect nonlinear interdependence structures among variables.

**Figure 7:**
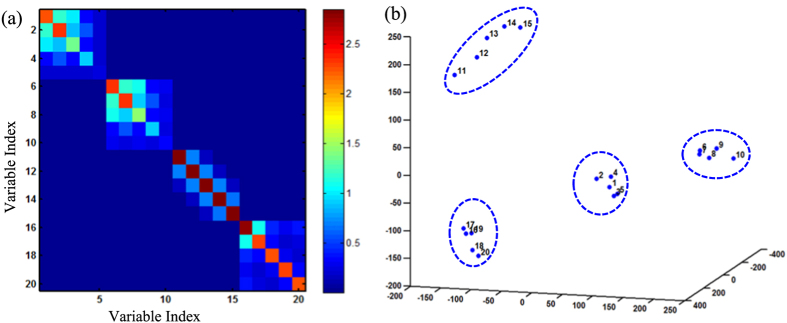
(**a**) Mutual information based dissimilarity matrix; (**b**) Dirichlet process clustering of simulated variables.

Figure 7a shows the mutual information based correlation matrix among variables that are computed from the simulated data set. The red color represents a higher nonlinear correlation, while the blue color indicates no interrelationships. Figure 7a shows significant nonlinear correlation within the simulated clusters. Also, variables from different clusters have little interrelationship. If we use Dirichlet process to cluster variables based on low-dimensional vectors embedded from the dissimilarity matrix of mutual information, four clusters of variables are distinctly separated in the space (see Fig. 7b). The simulation study shows that Dirichlet process models effectively cluster these 20 variables into 4 groups and identifies the underlying cluster structures of variables.

### Real-world Case Study

In the previous work, we characterized and represented 3-dimensional vectorcardiogram (VCG) signals using a sparse basis function model^26^. This sparse representation not only reduces large amounts of data to a limited number of model parameters, but also preserves the signal information. As opposed to the original data, this present paper will utilize parameters in basis function models as explanatory variables to further predict the myocardial infarctions. VCG signals are represented by *L* superposed basis functions in order to capture intrinsic characteristics of cardiac electrical activity as:


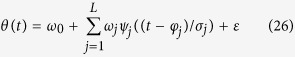


where 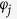 and *σ*_*j*_ are shifting and scaling factors, *ψ*_*j*_(·) are basis functions, and *ω*_*j*_ are weight factors, respectively. The objective is to optimize the sparse representation of 3D VCG signals:


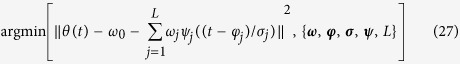


In order to identify a compact set of basis functions that minimize the representation error, the number of basis functions *L* is minimized and basis functions ***ψ*** are optimally placed. Model parameters ***ω***, 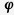, ***σ*** are adaptively estimated by “best matching” projections of VCG signals onto a dictionary of nonlinear basis functions. The optimization algorithms of a sparse basis function representation for spatiotemporal VCG signals were detailed in our previous work^26^.

In this present study, model parameters, i.e., weight, shifting, scaling factors and residuals, are extracted from the sparse basis function representation of VCG signals, and then are further utilized as explanatory variables for the identification of cardiac disorders (i.e., myocardial infarctions). The parameter set is {***ω***_3×*L*_, ***ϕ***_3×*L*_, ***σ***_3×*L*_} for *L* basis functions because there are 3 channels of signals in 3-lead VCG. Our previous study^26^ showed that modeling performance is greater than 99.9% goodness-of-fit with a parsimonious set of 20 basis functions for a variety of cardiac conditions. Hence, a total of 180 model parameters is adaptively estimated from the 3D VCG trajectory. In addition, we add other parameters in this present investigation, the overall feature matrix is:


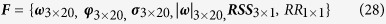


where |***ω***|_3×20_ are absolute values of weights, describing amplitudes of each basis function and indicating local strengths of a heartbeat. The residual sum of squares ***RSS***_3×1_ measure the discrepancy between model representation and VCG signals in each channel. The heart rate *RR*_1×1_ characterizes temporal beat-to-beat variations of cardiac electrical activity. Therefore, these 244 parameter-based features are used to represent the details of original VCG signals. Notably, the high-dimensional VCG signals are reduced into a parsimonious set of model parameters using the sparse representation without losing clinically important information.

A total of 388 (79 controls and 309 infarctions) 3-lead VCG signals, available in the PhysioNet Database^27^, are used in this investigation. These signals were digitized at 1 kHz sampling rate with a 16-bit resolution over a range of 16.384 mV. Our previous study showed that most of model-driven parameters (146 over 244 features) are statistically significant between healthy controls and diseased conditions, i.e., Kolmogorov-Smirnov (K-S) statistics are greater than critical value 0.17^28^. In addition, weight factors yield larger K-S statistics than other parametric features. However, the “curse of dimensionality” as well as the overfitting problems come out with a large number of predictors for the predictive modeling. Therefore, the lasso-penalized logistic regression model was utilized to shrink the number of predictors and further identify cardiac disorders (i.e., myocardial infarctions) in our previous study^28^.

**Figure 8:**
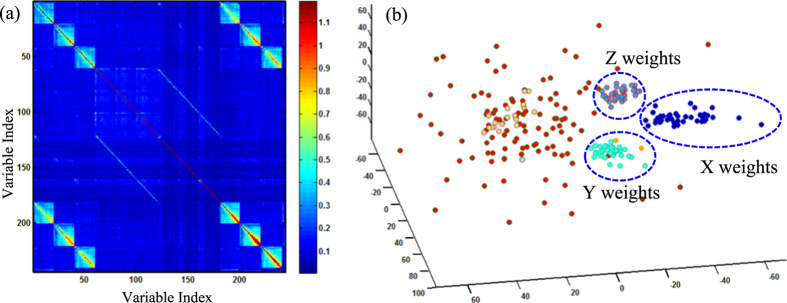
(**a**) Dissimilarity matrix based on mutual information measured between variables; (**b**) Dirichlet process clustering of model-based parametric features.

Nonetheless, our previous study^28^ focused on the relevancy between predictor and response variables, without specifically considering nonlinear interdependence structures among predictor variables. Prior research showed that the collinearity (i.e., large correlation between variables) leads to stability problems in predictive models (i.e., increased variances of estimation)^29^. The present paper further investigates the nonlinear correlations between variables and then identifies the cluster structures of variables for improving the predictive performance. Figure 8a shows the visualization of information-based dissimilarity matrix measured among variables. It may be noted that six groups of variables have stronger nonlinear relationships, i.e., ***ω***_3×20_ and |***ω***|_3×20_ as the weights and absolute weights of X, Y and Z-axis directions. However, few, if any, previous work has explicitly considered such relationships among variables in the process of predictive modeling. Moreover, weight factors ***ω***_3×20_ also have strong nonlinear correlation with the variables of absolute weights |***ω***|_3×20_. Without taking these nonlinear interrelationships into account, predictive models are sensitive to extraneous noises and are limited in the ability to provide an effective prediction of myocardial infarctions.

Figure 8b shows the nonparametric Dirichlet process for variable clustering of model-based parametric features. As shown in Fig. 8b, the Dirichlet process cluster all the variables into five groups based on the embedding features from the variable-to-variable dissimilarity matrix of mutual information. Three clusters are shown to be significant, i.e., weight and absolute weight variables of X, Y and Z-axis respectively. As a result, homogeneous variables are clustered into subset communities. It may be noted that the result of variable clustering is consistent with the prior knowledge and the variable-to-variable dissimilarity matrix of mutual information.

**Figure 9:**
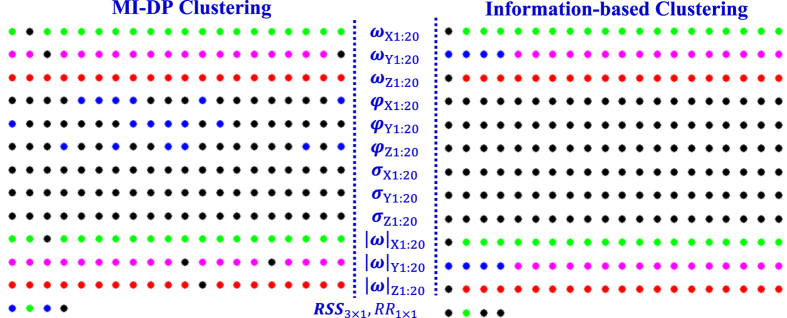
The results of variable clustering by (**a**) MI-DP clustering and (**b**) Information-based clustering with color coding.

**Figure 10:**
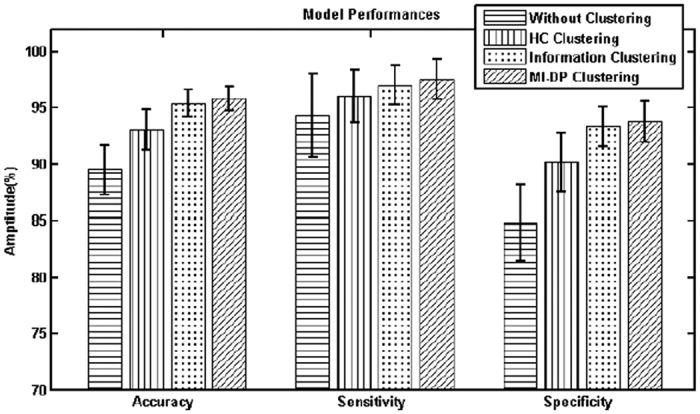
The comparison of averages and standard deviation of prediction performances in the real-world case study. “Without clustering”: lasso-penalized logistic regression model; “HC clustering”: group elastic-net model with hierarchical clustering using linear correlation measured between variables; “Information clustering”: information-based clustering^21^; “MI-DP clustering”: group elastic-net model with variable clustering using mutual information and Dirichlet Process Mixtures.

Figure 9 shows the results of variable clustering by our proposed algorithm and the information-based clustering. Note that there are 244 variables represented as color markers, and each marker with the same color represents the same cluster. Each row denotes a type of variables. For example, the first row of 20 markers is weight factors ***ω***_X1:20_ in the X-dimension of VCG signals. Figure 9a shows the clustering results for MI-DP clustering (also see Fig. 8b), while Fig. 9b shows the clustering results for the information-based clustering. It may be noted that information-based clustering was designed to cluster data samples rather than variables. We modified the original algorithm in ref. 21 for variable clustering. Because information-based clustering^21^ needs to predefine the number of clusters, we therefore use the same number of clusters identified by our proposed algorithm. Note that Fig. 9 shows there are slight differences in clustering results by MI-DP and information-based clustering. Figure 9b shows that a small portion of Y weights is not accurately clustered by information-based clustering. In addition, some variables such as shifting and scaling factors, and residuals are grouped together and cannot be well separated. As such, information-based clustering yields slightly inferior performance of predictive modeling in comparison with the proposed MI-DP approach (also see Fig. 10).

Figure 10 shows the comparison of prediction performances of different clustering procedures in the real-world case study. “Without clustering” represents the results from the lasso-penalized logistic regression model in our previous study^28^. “HC clustering” denotes the hierarchical clustering with linear correlation measured between variables. “Information clustering” is the information-based clustering from the literature^21^. “MI-DP clustering” is the proposed information theoretic approach for variable clustering using mutual information and Dirichlet Process Mixtures. As shown in Fig. 10, MI-DP clustering yields better performance than “Without clustering”. Note that MI-DP clustering improves the predictive accuracy from 89.50% to 95.84%, the sensitivity is improved from 94.33% to 97.56%, and the specificity is increased from 84.80% to 93.78%. In addition, MI-DP clustering yields smaller standard deviations of performance metrics (i.e., accuracy, sensitivity, and specificity) than “without clustering”. Similarly, the results of MI-DP clustering are better than “HC clustering” (i.e., accuracy 93.07%, sensitivity 96.05% and specificity 90.18%) and “Information clustering” (i.e., accuracy 95.38%, sensitivity 97.02% and specificity 93.33%). Experimental results showed that MI-DP clustering effectively delineates the nonlinear correlation structures among variables and further derive homogeneous groups of variables, thereby improving the prediction performance.

## Discussion and Conclusions

Advanced sensing and real-time data acquisition bring the proliferation of big data. This provides an unprecedented opportunity to move forward data-driven knowledge discovery. However, it is common that big data involves large amounts of variables with complex interdependence structures, which brings significant challenges on traditional modeling strategies. To tackle these challenges, variable selection and variable clustering are widely used in the literature. Nonetheless, variable selection focuses primarily on the relevancy between predictors and response variables, but does not explicitly consider the redundancy among variables. The variable clustering, on the other hand, focuses on the linear relevancy between variables. There is a need to develop new methodologies to improve the effectiveness and efficiency of variable clustering and predictive analytics.

The computational complexity of MI-DP clustering consists of three components, namely measure of mutual information, low-dimensional embedding, and DP variable clustering. First, mutual information is measured among *N*(*N* − 1)/2 pairs of variables. The computational complexity for one pair of variables is 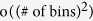, i.e., 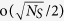. Hence, the complexity is approximately 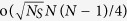. Second, the complexity of low-dimensional embedding is shown to be 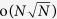 in the literature^30^. Third, the Dirichlet process allocates each variable to a cluster with a computational complexity of o(*N*). In the present case studies, there are not significant challenges in computational complexity. However, it is worth mentioning that a new research direction is to design efficient algorithms to compute the pairwise mutual information (MI) between all pairs of variables, which will significantly improve the performance of MI-DP approach for big data applications.

This paper presents a new information-theoretic approach for variable clustering and predictive modeling using Dirichlet process mixtures. This new methodology investigates both redundancy and relevancy among variables for improving the performance of predictive modeling. Both simulation and real-world case studies demonstrate that the proposed MI-DP clustering algorithm not only outperforms traditional methods (i.e., lasso-penalized variable selection and classical hierarchal clustering), but also identifies nonlinear interdependence structures among variables and further improves the performance of predictive modeling. The new methodology of MI-DP variable clustering is generally applicable for predictive modeling in many disciplines that involve a large number of highly-redundant variables. In the future work, we will also consider the integration of our proposed MI-DP clustering algorithm with co-clustering approach to investigate the nonlinear interdependence among subsets of both samples and variables.

## Additional Information

**How to cite this article**: Chen, Y. and Yang, H. A Novel Information-Theoretic Approach for Variable Clustering and Predictive Modeling Using Dirichlet Process Mixtures. *Sci. Rep.* **6**, 38913; doi: 10.1038/srep38913 (2016).

**Publisher's note:** Springer Nature remains neutral with regard to jurisdictional claims in published maps and institutional affiliations.

## Figures and Tables

**Figure 1:**
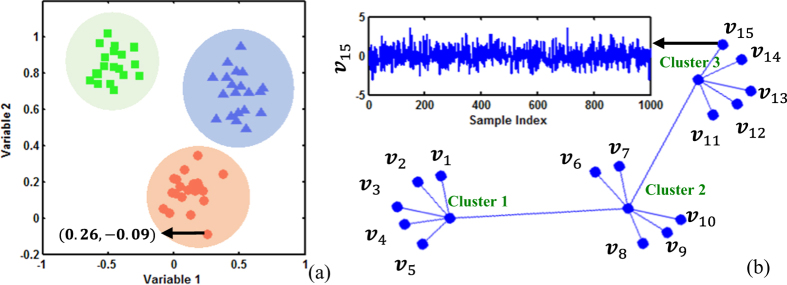
(**a**) Data clustering with each point representing a data sample; (**b**) Variable clustering with each point representing a variable.

**Table 1:** Four cluster of simulated variables.

Cluster #	1^st^ variable	2^nd^ variable	3^rd^ variable	4^th^ variable	5^th^ variable
1	***v***_1_	***v***_2_ = |***v***_1_|	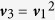	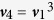	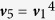
2	***v***_6_	***v***_7_ = |***v***_6_|	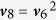	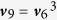	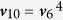
3	***v***_11_	***v***_12_ = ***v***_11_(*t* + 3)	***v***_13_ = ***v***_11_(*t* + 5)	***v***_14_ = ***v***_11_(*t* + 7)	*v*_15_ = *v*_11_(*t* + 9)
4	***v***_16_	***v***_17_ = ***v***_16_(*t* + 10)	***v***_18_ = ***v***_16_(*t* + 20)	***v***_19_ = ***v***_16_(*t* + 30)	***v***_20_ = ***v***_16_(*t* + 40)

Where ***v***_1_ and ***v***_6_ are independent standard normal variables, ***v***_11_ is a nonlinear variable sampled from logistic map ***v***_11_(*n* + 1) = 3.8***v***_11_(*n*)(1 − ***v***_11_(*n*)), ***v***_16_ is a second-order autoregressive variable that is nonlinearly coupled with *x*_*Lorenz*_, 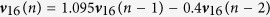 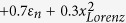, where 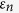 is Gaussian noise, *x*_*Lorenz*_ is the *x*-component of a Lorenz system: 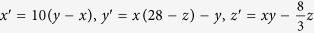 with time step 0.01. Each variable has a sample size of 1000.

## References

[b1] Verhoef P. C. & Donkers B. Predicting customer potential value an application in the insurance industry. Decision Support Systems 32, 189–199 (2001).

[b2] Chen Y. & Yang H. Sparse Modeling and Recursive Prediction of Space-time Dynamics in Stochastic Sensor Network. IEEE Transactions on Automation Science and Engineering 13, 215–226 (2016).

[b3] Chen Y. & Yang H. *Heterogeneous postsurgical data analytics for predictive modeling of mortality risks in intensive care units* (Proceedings of 2014 IEEE Engineering in Medicine and Biology Society Conference (EMBC), 2014). 10.1109/EMBC.2014.6944578 25570946

[b4] Yang H. & Kundakcioglu E. Healthcare Intelligence: Turning Data into Knowledge. Intelligent Systems, IEEE 29, 54–68 (2014).

[b5] Ward J. H. Hierarchical Grouping to Optimize an Objective Function. Journal of the American Statistical Association 58, 236–244 (1963).

[b6] Airoldi E. M., Blei D. M., Fienberg S. E. & Xing E. P. Mixed Membership Stochastic Blockmodels. Journal of Machine Learning Research 9, 1981–2014 (2008). 21701698 PMC3119541

[b7] Acharya A. . In ECML PKDD (eds Appice A. .) 283–299 (2015).

[b8] Zhou M. Infinite Edge Partition Models for Overlapping Community Detection and Link Prediction. *In Proceedings of AISTATS* **38** (2015).

[b9] Blei D. M., Ng A. Y. & Jordan M. I. Latent Dirichlet Allocation. Journal of Machine Learning Research 3, 993–1022 (2003).

[b10] Blei D. M. & McAuliffe J. D. Supervised topic models. Advances in Neural Information Processing Systems (NIPS), 121–128 (2007).

[b11] Zhu J., Ahmed A. & Xing E. P. MedLDA: Maximum Margin Supervised Topic Models. Journal of Machine Learning Research 13, 2237–2278 (2012).

[b12] Zhu J., Chen N., Perkins H. & Zhang B. Gibbs Max-Margin Topic Models with Fast Sampling Algorithms. In Proceedings of ICML 28 (2013).

[b13] Cheng Y. & Church G. M. Biclustering of Expression Data. *In* Proceedings of ISMB 8, 93–103 (2000). 10977070

[b14] Dhillon I. S. Co-clustering documents and words using bipartite spectral graph partitioning. *In* Proceedings of ACM SIGKDD, 269–274 (2001).

[b15] Dhillon I. S., Mallela S. & Modha D. S. Information-theoretic co-clustering. *In* Proceedings of ACM SIGKDD, 83–89 (2003).

[b16] Deodhar M. & Ghosh J. SCOAL: A Framework for Simultaneous Co-Clustering and Learning from Complex Data. ACM Transactions on Knowledge Discovery from Data 4, 11–31 (2010).

[b17] Pearson K. Notes on regression and inheritance in the case of two parents. Proceedings of the Royal Society of London 58, 240–242 (1895).

[b18] Fraser A. M. & Swinney H. L. Independent coordinates for strange attractors from mutual information. Phys. Rev. A 33, 1134–1140 (1986). 10.1103/physreva.33.1134 9896728

[b19] Kinney J. B. & Atwal G. S. Equitability, mutual information, and the maximal information coefficient. PNAS 111, 3354–3359 (2014). 24550517 10.1073/pnas.1309933111 PMC3948249

[b20] Reshef D. N. . Detecting Novel Associations in Large Data Sets. Science 334, 1518–1524 (2011). 22174245 10.1126/science.1205438 PMC3325791

[b21] Slonim N., Atwal G. S., Tkačik G. & Bialek W. Information-based clustering. PNAS 102, 18297–18302 (2005). 16352721 10.1073/pnas.0507432102 PMC1317937

[b22] Jain A. K. Data clustering: 50 years beyond K-means. Pattern Recognition Letters 31, 651–666 (2010).

[b23] Le T. Q., Cheng C., Sangasoongsong A., Wongdhamma W. & Bukkapatnam S. T. S. Wireless wearable multisensory suite and real-time prediction of obstructive sleep apnea episodes. IEEE Journal of Translational Engineering in Health and Medicine 1, 2700109 (2013). 27170854 10.1109/JTEHM.2013.2273354 PMC4819230

[b24] Blei D. M. & Jordan M. I. Variational inference for Dirichlet process mixtures. Bayesian Analysis 1, 121–144 (2006).

[b25] Zou H. & Hastie T. Regularization and variable selection via the elastic net. J. R. Statist. Soc. B 67, 301–320 (2005).

[b26] Liu G. & Yang H. Multiscale adaptive basis function modeling of spatiotemporal cardiac electrical signals. IEEE Journal of Biomedical and Health Informatics 17, 484–492 (2013). 24235119 10.1109/JBHI.2013.2243842

[b27] Goldberger A. L. . PhysioBank, physiotoolkit, and physionet: Components of a new research resource for complex physiologic signals. Circulation 23, e215–e220 (2000). 10.1161/01.cir.101.23.e215 10851218

[b28] Liu G., Kan C., Chen Y. & Yang H. Model-driven parametric monitoring of high-dimensional nonlinear functional profiles. Automation Science and Engineering (CASE), 2014 IEEE International Conference. 722–727 (2014).

[b29] Nas T. & Mevik B. H. Understanding the collinearity problem in regression and discriminant analysis. Journal of Chemometrics 15, 412–426 (2001).

[b30] Morrison A., Ross G. & Chalmers M. Fast multidimensional scaling through sampling, springs and interpolation. Information Visualization 2, 68–77 (2003).

